# 

**Published:** 1847-12

**Authors:** 


### WESTERN JOURNAL

OF

### MEDICINE AND SURGERY.

EDITORS.

DANIEL DRAKE, M. D.,

PROFESSOR OF PATHOLOGY AND THE PRACTICE OF MEDICINE IN THE UNIVERSITY OF LOUISVILLE.

LUNSFORD P. YANDELL, M. D.,

PROFESSOR OF CHEMISTRY AND PHARMACY IN THE SAME.

THOMAS W. COLESCOTT, M. D.

UNIVERSITY OF LOUISVILLE.

The classes in both the Law and Medical Department of the University of Louisville are larger than they were last winter. The annual circular, to be issued the first of January, will exhibit remarkable prosperity in the institution. We may say, in one word, that the spacious rooms in the Medical College are full.

---

BOOKS RECEIVED.

From the publishers, Messrs. Grigg & Elliott, we have received a copy of Woos' Practice; and from Messrs. Lea & Blanchard, copies of Bartlett on Fever, and Carpenter's Physiology, all of which will be noticed more at length in an early number. For sale at the book stores of Mrs. Maxwell and Mr. Prescott, in this city.

---

ODD NUMBERS WANTED.

The publishers of this Journal will pay fifty cents each for the numbers for April, October, and December, 1845. Any one having those numbers to spare, will oblige the publishers by remitting them forthwith by mail.

### INDEX TO VOL. VIII.

[NEW SERIES.]

Abstract, Ranking's............276
Adulterations in medicine.......181
Affections, cerebral............376
Alcohol, test for water in........24
Almanac, Medical..............442
Alum in pertussis ..............348
Alum, insufflations of..........254
Aneurism, acupuncture in.........23
Aneurism, treatment of.........221
Appointments, medical..........461
Autopsy .........................29
Agassiz's lecture..............535

Baldwin on quinine............426
Bellingham on aneurism........221
Bladder, action of strychnine on.30
Blood, state of in scurvy.......388
Boling on black death..........209
Boling on fistula in ano........301
Books and correspondents ......368
Botany, medical..................58
Brain, structure of.............379
Breast, cancer of.................33
Burmah, medicine in............515

Canada, ship-fever in...........260
Cancer of breast ..................33
Carroll on cancrum oris ..........93
Case of sarcocele..................40
Case of syphilis.................200
Cerebral affections.............376
Chelius on surgery ..............436
Chemistry, Silliman's...........367
Children, four at a birth........483

City, health of..................199
Clinical cases...................479
Clinical medicine...............368
Clinical Reports...........42, 479
Combe, Dr. Andrew............449
Convention, Medical.. 82, 183, 462
Convulsions ....................479
Copper, arsenite of.............346
Croup cured .....................81
Croup, treatment of ............258
Catarrh of the uterus...........463
Croup, Fee's case of............493
Cholera, secretions in..........515

Death, autopsy after..............29
Death, black, in Florence.......209
Death, new sign of ..............21
Death of Lisfranc.................90
Death of Prof. Warner............90
Delirium tremens ...............219
Diseases of Indiana................1
Diseases from the use of tobacco.63
Donnell's case of labor..........483
Dowling on diseases of Indiana...1
Drake on ship-fever ............358
Dysmenorrhea ..................245
Disinfecting fluid...............545
Death from over-eating.........487
Delirium tremens, ether in....537
Dawson on puerperal fever....512
Diamond into coke..............529

Effects of temperature ........257
Elbow, luxation of.............279

Electricity and nervous fluid ....21
Electricity, new mode of applying.24
Emphysema, opium in..........25
Epistaxis, treatment of........254
Epsom salts, substitute for.....350
Ergotine in hemorrhage.........20
Ether, effects of..............153
Ether, effects on the blood......20
Ether injected into bloodvessels..22
Ether in delirium tremens......537
Ether in labor ................71
Etherization...................368
Ether, sulphuric ..............423
Ether vapor................70, 78
Ethics, medical ...............165
Eupatorium perfoliatum........347
Excision of tonsils.............41
Eye, diseases of...............58

Fistula in ano.................301
Four children at a birth .......483
Fee's case of croup............493
Fevers................512, 548

Geological survey .............180
Geology of Kentucky ..........310
Gordon's case, delirium tremens.219
Greenley on tetanus ...........418

Hare Prof. resignation of........89
Harris, vesico-vaginal fistula....217
Headache, varieties of ....250, 275
Health of Louisville...........179
Hydatids of spinal cord ........26
Hastings Dr., death of.........531
Hydrocyanic acid, death from..540

Injurious symptoms from ether..78
Instrumental labor, ether in.....71
Intestinal secretions in cholera..515

Journal, postage on this .......184

Kentucky, geology of.........310
Kearney Dr., death of.........532

Labor, ether in instrumental ....71
Labor premature ..............443
Larynx cauterized..............81
Latent pneumonia.............252
Latham's Clinical Medicine....368
Lectures of M. Agassiz........535
Lecture terms, length of .......182
Lisfranc, death of ..............90
Louisville Univ'y 184, 276, 368, 550
Luxation of elbow.............379
Ledoyen's disinfectant.........545
Liver, extirpation of...........549
Light on plants...............534
Lightning rods................539

Medical Almanac ..............442

Medical appointments..........461

Medical changes ..............276

Medical character of Saratoga...66

Medical Convention ...82, 183, 462

Medical matters and men .19, 28, 185, 277, 369, 463

Medical reform.......192, 274, 462

Medical services...............262

Medicine, adulterations in .....181

Medicine, Institutes of ........440

Medicine, veterinary............90

Mendenhall's Vade-Mecum.....62

Meningitis ....................128

Midwifery, ether in ............70

Miscarriage, prevention of .....443

Moorman on Virginia springs ...60

Mott's Velpeau .................57

Murphy's parturition ..........181

Murphy on parturition.........497

Medicine in Burmah...........515

Medical heroism..............530

Neil's Outlines ...............442
Neuralgia....................454
Nitrous oxide gas.............343

Obituary notice ..............449
Ohio Medical Convention......462
Operation, painless .............91
Operation, surgical ...........346
Operative Surgery..............57
Opium in emphysema...........25
Osborne's cases...............487

Paine's Institutes..............440
Painless operation..............91
Parturition, Murphy's.........181
Pertussis, alum in.............348
Physic, Principles and Practice.441
Pleurisy......................475
Pneumonia, latent............252
Poisoning by arsenite of copper.346
Postage on Journal ...........184
Potassium, bromide and iodide of.254
Pump, improved .............180

Pleurisy acute................475
Purpura hemorrhagica........488
Pseudo-membranous croup....493
Puerperal fever...............512
Plants, light on the growth of..534

Quinine, mode of administering.262
Quinine, poisonous properties of.426

Ranking's Abstract............276
Reform, medical.....192, 274, 462

Salt-wells, temperature of......92
Saratoga Springs...............66
Sarcocele......................382
Sarcocele, case of..............40
Sayre's case...................485
Scrotal tumor..................485
Scurvy, state of blood in......368
Ship-fever in Canada..........260
Ship-fever in New York.......396
Silliman's Chemistry..........367
Smallpox, treatment of........151
Spinal cords, hydatids of.......26
Stone, solvent for...............20
Structure of the brain..........389
Strychnine on the bladder.......30
Student's Vade-Mecum...........62
Substitute for Epsom salts.....350
Scarlatina......................493
Sulphur rains..................544
Surgery, system of.............436
Surgical operation.............346
Survey, geological.............180
Sutton on medical reform.....403
Symptoms, injurious, from ether.78
Syphylis, case of..............200
Syphilis, treatment of.........254

Temperature, changes of......257
Temperature of salt-wells......92
Term of utero-gestation.......349
Testicle, scirrhous............343
Testicle, venereal.............385
Tetanus, case of..............418
Test for water in alcohol.......24
Thoracentesis.................475
Tic douloureux...............454
Tobacco, immoderate use of....63
Tonsils, excision of.............41
Traveling letters..263, 270, 351, 358, 455
Treatment of croup...........258
Typhus and typhoid fever.....548

Union by first intention.......443
Use of eupatorium perfoliatum.347
Utero-gestation, term of.......349
Uterus, catarrh of.............548
University of Louisville.......550

Variola, vaccinia, varioloid, etc.253
Veins and lymphatics..........442
Venereal testicle..............385
Vesico-vaginal fistula..........217
Veterinary medicine............90
Virginia springs.................60

Warner, death of Prof.........90
Water-pumps, improved......180
Watson's Principles and Pract..441
Wendel on ship-fever.........396
Wendel's Reports.........42, 479
Wharton on ether.............423
Worms in the heart of a dog...491

Yandell & Shumard on geology.310
Yandell on medical matters and men..19, 128, 185, 277, 369, 463

To Subscribers. The present volume of this Journal closes with this number. We trust that subscribers in arrears will at once remit, and that those who have paid for 1847 will forward the subscription for 1848. Subscribers will perceive from the published lists that our receipts have been very light, notwithstanding that we have paid large sums for contributions and correspondence. We hope that they will now respond by prompt and liberal remittances.

PRENTICE & WEISSINGER.

*Receipts of Medical Journal for October and November.*

| | |
|---|---|
| Dr. W. H. Stockwell, Patoka, Ind | $5 00 |
| S. B. Hobbs, Reeves, Mo | 5 00 |
| L. Sullivan, Mortonsville, Ky | 5 00 |
| J. D. Jones, Circleville, Ohio | 20 00 |
| M. Hoag, Lodi, Ohio | 20 00 |
| O'Haver, Carlisle, Ind | 10 00 |

TRAVELING AGENTS FOR THE WESTERN JOURNAL OF MEDICINE AND SURGERY.

Jno. F. Johnston and Jas. H. Stevenson, Kentucky—C. W. James, Ohio, Indiana, Illinois, Missouri, and Michigan—Geo. C. Brett, Tennessee—Wm. H. Weld & Son, Mississippi and Louisiana.

The Western Journal of Medicine and Surgery is published monthly by the undersigned, at the corner of Main and Fifth streets, Louisville, at $5 per annum, payable in advance. Each number contains 96 pages, making two volumes in the year of more than 550 pages each.

Contributions to its pages by the Physicians of the Valley of the Mississippi, addressed to the Editors, are respectfully solicited.

Letters on business, to be addressed, postage paid, to the publishers.

*January*, 1844. PRENTICE & WEISSINGER.

